# Standardization of zebrafish drug testing parameters for muscle diseases

**DOI:** 10.1242/dmm.050339

**Published:** 2024-01-18

**Authors:** Muthukumar Karuppasamy, Katherine G. English, Clarissa A. Henry, M. Chiara Manzini, John M. Parant, Melissa A. Wright, Avnika A. Ruparelia, Peter D. Currie, Vandana A. Gupta, James J. Dowling, Lisa Maves, Matthew S. Alexander

**Affiliations:** ^1^ Division of Neurology, Department of Pediatrics, University of Alabama at Birmingham and Children's of Alabama, Birmingham, AL 35294, USA; ^2^ Graduate School of Biomedical Science and Engineering, University of Maine, Orono, ME 04469, USA; ^3^ School of Biology and Ecology, University of Maine, Orono, ME 04469, USA; ^4^ Child Health Institute of New Jersey and Department of Neuroscience and Cell Biology, Rutgers, Robert Wood Johnson Medical School, New Brunswick, NJ 08901, USA; ^5^ Department of Pharmacology and Toxicology, University of Alabama at Birmingham Heersink School of Medicine, Birmingham, AL 35294, USA; ^6^ Department of Pediatrics, Section of Child Neurology, University of Colorado at Anschutz Medical Campus, Aurora, CO 80045, USA; ^7^ Department of Anatomy and Physiology, School of Biomedical Sciences, Faculty of Medicine Dentistry and Health Sciences, University of Melbourne, Melbourne, Victoria 3010, Australia; ^8^ Centre for Muscle Research, Department of Anatomy and Physiology, University of Melbourne, Melbourne, Victoria 3010, Australia; ^9^ Australian Regenerative Medicine Institute, Monash University, Clayton, Victoria 3800, Australia; ^10^ EMBL Australia, Victorian Node, Monash University, Clayton, Victoria 3800, Australia; ^11^ Division of Genetics, Department of Medicine, Brigham and Women's Hospital, Harvard Medical School, Boston, MA 02115, USA; ^12^ Division of Neurology, The Hospital for Sick Children, Toronto, Ontario M5G 1X8, Canada; ^13^ Department of Paediatrics, University of Toronto, Toronto, Ontario M5G 1X8, Canada; ^14^ Program for Genetics and Genome Biology, The Hospital for Sick Children, Toronto, Ontario M5G 0A4, Canada; ^15^ Department of Molecular Genetics, University of Toronto, Toronto, Ontario M5G 0A4, Canada; ^16^ Center for Developmental Biology and Regenerative Medicine, Seattle Children's Research Institute, Seattle, WA 98101, USA; ^17^ Department of Pediatrics, University of Washington, Seattle, WA 98195, USA; ^18^ UAB Center for Exercise Medicine, University of Alabama at Birmingham, Birmingham, AL 35294, USA; ^19^ Department of Genetics, University of Alabama at Birmingham, Birmingham, AL 35294, USA; ^20^ Civitan International Research Center, University of Alabama at Birmingham, Birmingham, AL 35294, USA; ^21^ UAB Center for Neurodegeneration and Experimental Therapeutics (CNET), Birmingham, AL 35294, USA

**Keywords:** Zebrafish, Drug discovery, Standardization, Drug screening parameters, Drug library

## Abstract

Skeletal muscular diseases predominantly affect skeletal and cardiac muscle, resulting in muscle weakness, impaired respiratory function and decreased lifespan. These harmful outcomes lead to poor health-related quality of life and carry a high healthcare economic burden. The absence of promising treatments and new therapies for muscular disorders requires new methods for candidate drug identification and advancement in animal models. Consequently, the rapid screening of drug compounds in an animal model that mimics features of human muscle disease is warranted. Zebrafish are a versatile model in preclinical studies that support developmental biology and drug discovery programs for novel chemical entities and repurposing of established drugs. Due to several advantages, there is an increasing number of applications of the zebrafish model for high-throughput drug screening for human disorders and developmental studies. Consequently, standardization of key drug screening parameters, such as animal husbandry protocols, drug compound administration and outcome measures, is paramount for the continued advancement of the model and field. Here, we seek to summarize and explore critical drug treatment and drug screening parameters in the zebrafish-based modeling of human muscle diseases. Through improved standardization and harmonization of drug screening parameters and protocols, we aim to promote more effective drug discovery programs.

## Teaser

**Summary:** We propose a standardization of criteria for drug screening and evaluation in zebrafish muscle disease models, with the goal of improving experimental rigor, reproducibility and translation.

## Introduction

Skeletal muscle disorders, such as muscular dystrophies and myopathies, predominantly target the skeletal and cardiac muscles, peripheral nerves, and respiratory and smooth muscles. The consequences of these neuromuscular disorders (NMDs) often include skeletal muscle wasting, muscle degeneration, loss of muscle function, lack of mobility, impaired breathing, cardiomyopathy and diaphragm abnormalities (Emery, 1991; Dubowitz, 1978; Monaco et al., 1986; Hoffman et al., 1987; Morrison, 2016; Mary et al., 2018). Recent approaches, including gene editing, gene replacement therapy, cell therapy and RNA-based technologies such as antisense oligonucleotides, have made progress toward clinical trials that could be beneficial in ameliorating certain NMDs (Findlay and Weihl, 2022; Lim and Yokota, 2023; Fatehi et al., 2023). However, these approaches have significant challenges, such as mutation-specific targeting and attaining efficient and body-wide delivery. These ongoing challenges make it imperative to continue to pursue and identify novel small-molecule drugs for muscle disorders.

One of the most practical approaches for drug discovery for muscle disorders is to utilize the zebrafish for screening chemical compounds. However, chemical screening in zebrafish requires standardization to advance therapeutic molecules for the end goal of treating patients. Our aim with this Special Article is to provide guidelines and standards for using zebrafish models in drug evaluations for muscle disease. Similar to previous guidelines established for the *mdx* mouse model for Duchenne muscular dystrophy (DMD) (Willmann et al., 2012), we propose these standards to improve the power, rigor and reproducibility of preclinical zebrafish studies for advancing therapies for patients with muscle disease.

## Rationale for the use of zebrafish to evaluate drugs for muscle diseases

**Fig. 1.**
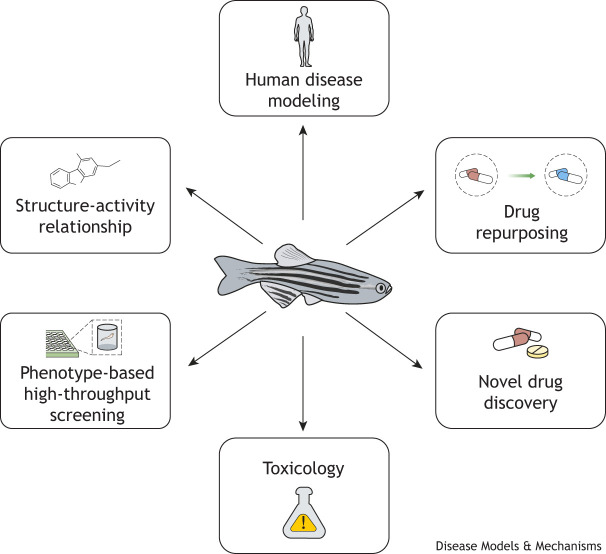
**Applications of the zebrafish model for drug discovery and muscle disease research.** Schematic highlighting multiple uses for the zebrafish model, including human disease modeling, repurposing of existing clinically approved drugs, discovery of novel drug compounds, toxicology, phenotype-based high-throughput screening, and characterization of the relationship between the structure of a molecule and its (therapeutic) activity.

The zebrafish (*Danio rerio*) is an advantageous vertebrate model for preclinical drug screening and discovery (Widrick et al., 2019; Patton et al., 2021). Compared to mammals, zebrafish possess many advantages, including their transparent early embryonic development, a high number of offspring, ease of genetic manipulation, inexpensive husbandry and the absorption of small molecules or chemical moieties through their skin and gills. The zebrafish genome is 70% identical to that of humans, and approximately 80% of genes associated with human disease are conserved between the two species (Howe et al., 2013). The zebrafish is an established model in developmental biology and disease modeling, and an emerging tool for translational studies to the clinic. High-throughput and high-content phenotypic screening, as well as repurposing of existing regulatory agency-approved drugs, rely on the versatile nature of the zebrafish model (Fig. 1).

Zebrafish models have been established for various human disorders, such as congenital heart defects (Zhao et al., 2019c), cancers (Cagan et al., 2019; Barriuso et al., 2015), neurological disorders (Kalueff et al., 2013; Griffin et al., 2018), liver dysfunction (Goessling and Sadler, 2015), kidney disease (Cirio et al., 2015) and blood disorders (Rissone and Burgess, 2018). In addition, there are several well-characterized zebrafish models of many diverse human skeletal NMDs (Table 1). In particular, zebrafish are a well-characterized animal model for DMD. A zebrafish *dmd*-deficient strain, also known as *sapje*, has a nonsense mutation in exon 4 (Bassett et al., 2003; Berger et al., 2010; Granato et al., 1996). Homozygous *sapje* embryos exhibit degenerative muscle lesions by about 3 days post fertilization (dpf) and also show many aspects of human DMD pathology, such as skeletal muscle fibrosis and inflammation (Bassett and Currie, 2004; Berger et al., 2010). Many zebrafish NMD models, including *dmd*-deficient zebrafish, have been used in chemical screens that have led to the identification of promising therapeutic compounds and new biomarkers (Table 1). These studies have highlighted the potential of zebrafish for identifying new therapeutic compounds and targets, as well as for understanding the molecular mechanisms behind muscle diseases (Widrick et al., 2019).

**Table 1.**
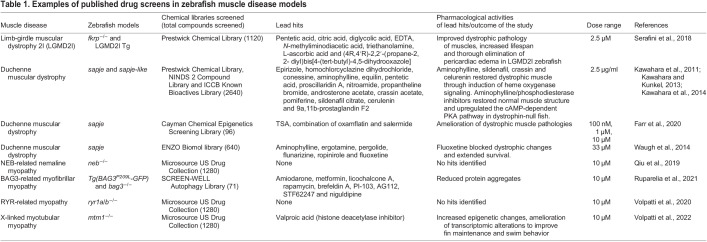
Examples of published drug screens in zebrafish muscle disease models

A key rationale behind small-molecule screening in zebrafish is the eventual translation of pharmacological and biological insights. To optimize translational efforts, several important parameters in drug screening methodologies should be documented and standardized, including drug dosing, aspects of zebrafish husbandry and how phenotypic endpoints are assessed. Below, we discuss drug screening parameters and standards pertaining to muscular disease drug discovery research in zebrafish.

## Recommended standards for drug screening parameters in zebrafish

**Fig. 2.**
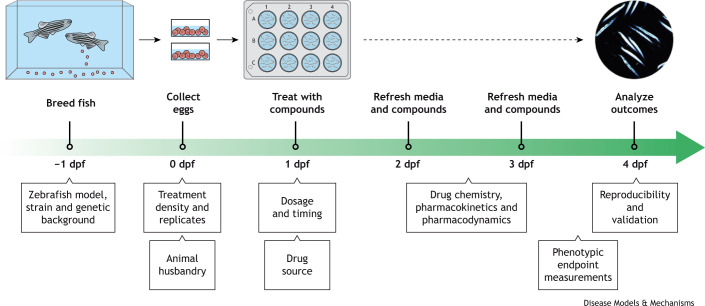
**Standard zebrafish drug treatment design.** The timeline shows a standard zebrafish drug treatment workflow, as described in many of the studies listed in Table 1. Below the timeline are several variables and parameters that need to be considered when conducting drug screening and small-molecule testing in zebrafish. Adult fish of the genetic background of choice are bred, and at 1 day post fertilization (dpf), embryos are treated with an appropriate dose of the drug (or control compound). Media containing the drug are changed each day, and the typical duration of exposure lasts 3 days. At 4 dpf, muscle integrity is examined via a number of available assays, as described in this Special Article. Notably, the duration of drug treatment and outcomes examined can be altered depending on the specific disease/model of interest.

Fig. 2 illustrates a standard zebrafish drug treatment design, employed in several of the studies listed in Table 1, and shows several variables and parameters that must be considered when conducting drug screening and small-molecule testing in zebrafish. Table 2 provides some examples where these parameters have been addressed in zebrafish NMD drug studies. In the following sections, we describe how these parameters can and should be standardized for zebrafish-based drug screening.

**Table 2.**
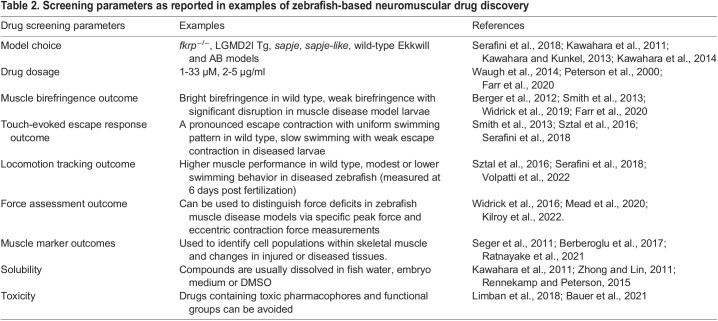
Screening parameters as reported in examples of zebrafish-based neuromuscular drug discovery

### Zebrafish strain and genetic background

One of the first parameters to consider is the specific mutant strain or genetic background to be used. Careful consideration should be given towards whether the zebrafish model is a spontaneous mutant, genomic DNA-edited mutant, morpholino-induced knockdown (morphant), transgenic line or one with gene overexpression. Spontaneous mutants and genomic DNA-edited mutants have the advantages of transgenerational stability at a particular genetic locus with predicted Mendelian ratios of mutant offspring and are most commonly used for drug screening purposes. Less commonly used models in zebrafish muscle drug screens are morphants, which usually result in transient knockdown of the expression of the gene of interest and may have off-target transcriptional effects (Stainier et al., 2017; Kok et al., 2015). Using overexpression models, usually generated by injecting mRNA, can result in reduced reproducibility due to injection variability and differences in levels of target protein expression between larvae. Consideration for these variables and the experimental question should be noted prior to conducting the drug testing.

Most zebrafish drug screens are conducted in mutant lines on the *AB* or *Tübingen* strain backgrounds, as most mutant lines have been generated via *N*-ethyl-*N*-nitrosourea (ENU)-induced mutagenesis or targeted genomic editing in these strain backgrounds (Crim and Lawrence, 2021). In NMDs such as DMD, genetic modifiers have been shown to play key roles in ambulation, cardiomyopathy, pulmonary function and, importantly, steroidal drug responses (Bello et al., 2015, 2020; Vianello et al., 2017; Barp et al., 2015). Therefore, to accurately interpret the phenotypic outcomes of a drug screen and to account for any potential contributions of genetic modifiers, it is important to report zebrafish strain background, if known, or whether a mixed background was used. Validating drug treatments using alternative alleles of the same gene to confirm responses is also strongly encouraged to reduce genetic background- or allele-specific effects. For example, the *sapje* (*dmd^ta222a^*) allele causes severe muscle pathology in homozygous mutants, and *sapje-like* (*dmd^cl100^*), which is an alternative allele with a dystrophin exon 62 splice junction variant, also results in severe muscle pathologies in homozygotes (Bassett et al., 2003; Guyon et al., 2009) and could be used for validation of initial drug screenings performed in the *sapje* fish. Additionally, three other *dmd-*null alleles were identified in a non-complementation screen of newly generated and existing mutants, all of which resulted in similar severe muscle pathologies in homozygotes (Berger et al., 2011). The existence of these multiple alleles was critical in testing therapeutic approaches such as exon skipping and the efficacy of drugs, such as ataluren, which has been proposed as a treatment for DMD (Berger et al., 2011, 2020). Testing the effects of lead drug compounds on multiple mutant strains is thus highly useful in validating drug responses in a disease-relevant context. If available to the investigator, the use of multiple zebrafish genetic lines harboring different genetic variants of the same gene for experimental validation is highly encouraged.

### Zebrafish embryo husbandry and dechorionation

Zebrafish drug studies are typically carried out using animals at embryonic (up to 3 dpf) and early larval stages (Fig. 2, Table 1). Thus, several husbandry variables need to be considered. Prior to 5-7 dpf, zebrafish embryos do not require feeding but do require a chemically balanced growth environment. There are several ‘standard’ zebrafish embryo medium recipes, such as E3 embryo medium, that can be found in The Zebrafish Book (Westerfield, 2000). Variations of zebrafish embryo media exist, and some investigators choose to use standard fish system water (see references in Table 1). As drug treatments are typically applied to zebrafish embryos through absorption from the embryo medium, variations in salts, pH or impurities might affect drug stability in the medium and their absorption and thus drug treatment results. Although we do not recommend a particular standard recipe for a zebrafish embryo medium here, the exact recipe used should be reported for all drug treatment studies. Furthermore, the embryonic medium may be supplemented with chemicals such as methylene blue, an antifungal agent, or 1-phenyl 2-thiourea (PTU), which is frequently used to inhibit pigment formation to improve live imaging. However, it is important to note that these may alter downstream pathways, such as autophagy activation in the case of PTU, subsequently confounding phenotype severities and the efficacy of any drugs being tested (Chen et al., 2021). The use of PTU or any other supplementary chemicals during embryo rearing should therefore be reported, and appropriate controls, including non-PTU-treated embryos, should be included. Moreover, there are alternative strategies for eliminating pigment to allow for live imaging of older embryos and larvae. For example, the *casper* mutant line harbors mutations in *mitfa* and *mpv17*, which are involved in melanin production (White et al., 2008; D'Agati et al., 2017). However, this approach can introduce complex genetics and potential genetic background issues, and some spawning issues with the *casper* line have been reported (Brantley and Lessman, 2019).

The optimal density for raising zebrafish embryos has been reported as 25-50 embryos/100 ml (Westerfield, 2000). However, zebrafish embryos are typically raised at 50-100/50 ml. Although overcrowding can lead to asynchronous and sub-optimal development (Kimmel et al., 1995), optimal density may not be feasible for all drug screening approaches or when testing scarce or expensive compounds. Examples of previous zebrafish *dmd* drug screens have used densities of 20 embryos/0.25 ml in 48-well plates (Kawahara et al., 2011) to 25 embryos/3 ml in 12-well plates (Farr et al., 2020). The size of the dishes used and drug solubility may also play a factor in determining the desired density for the zebrafish NMD drug screen (Castranova et al., 2011). Drug-containing embryo growth medium should be refreshed daily to alleviate any effects of overcrowding on optimal growth. In addition to fresh embryo medium, zebrafish require a temperature of 28.5°C for optimal growth and standardized staging (Kimmel et al., 1995). Embryo density, medium changes and growth temperature are all variables that should be reported for drug treatment experiments.

Dechorionation of the embryos is also crucial for drug testing in zebrafish embryos. The chorion serves as a protective barrier against pathogens and toxins but can impede drug penetration if not removed prior to drug treatments of embryonic zebrafish (Creton, 2004; Pelka et al., 2017). Dechorionation can be performed manually using fine forceps or by placing the embryos in a medium containing pronase, a commercial mixture of proteases. This is usually supplied at concentrations between 0.5-2 mg/ml with gentle agitation (Hasegawa et al., 2023). Pronase must be rinsed off once the chorion has been lysed to avoid damaging the embryos, and the dechorionated embryos are placed into fresh embryo medium or directly into the drug solution. Dechorionation is therefore another essential step to be standardized prior to drug testing and the methodology should be reported.

### Post-embryonic diet and other animal husbandry variables

Although most drug studies in zebrafish NMD models use embryonic-staged animals, in some cases, it is necessary to treat and examine outcomes well beyond the embryo stage to study drug treatment effects on long-term survival (Kawahara et al., 2011; Waugh et al., 2014; Serafini et al., 2018). Post-embryonic-stage experiments introduce the critical variable of diet. Zebrafish larval nutrition can affect musculoskeletal development (Printzi et al., 2023). Additionally, increasing evidence shows that zebrafish diet can have lasting impacts on both parental and offspring health and growth rate (Siccardi et al., 2009). Thus, animal diets and other post-embryonic husbandry variables can be important parameters to consider for zebrafish NMD drug studies. Unfortunately, there is no universally accepted zebrafish standard reference diet, such as that which has been developed for rodent models (AINCSNS, 1977; Watts and D'Abramo, 2021). Feeding of zebrafish larvae is usually initiated when they are placed onto a water-flow system at 5-7 dpf, at which time they may be given a diet of commercial dry food, live animals such as rotifers, live brine shrimp or paramecia, or a combination of these (Lawrence et al., 2016). Diets are often adjusted as zebrafish grow, and adult zebrafish are commonly fed a combination of commercial dry food and live brine shrimp (Lawrence et al., 2016). The timing of diet introduction and its composition should be reported as part of standard practice for all zebrafish experimentation (Fowler et al., 2019). Efforts toward developing a standard reference diet for zebrafish, similar to those for rodent models, are needed for optimal reproducibility of zebrafish research (Watts and D'Abramo, 2021).

Additional husbandry variables, including feeding times, light-dark cycles and housing densities, can substantially influence zebrafish growth rates (Lawrence et al., 2012; Villamizar et al., 2014). Any changes in aquaculture system homeostasis, diet, pathogen surveys or unexplained deaths should be monitored as part of standard zebrafish facility maintenance. Although the exact impact of these variables remains unclear, there is a growing body of evidence that many of these variables play key roles on zebrafish growth and reproductive rates and may potentially influence drug screening outcomes (Fowler et al., 2019). These zebrafish husbandry variables should be recorded and reported for reproducibility and standardization efforts of the field.

### Animal treatment density and replicates

Achieving statistical power in drug screens or drug treatment tests is essential and is based on adequate numbers of animals. To determine the number of zebrafish and replicates needed, a power analysis should be performed for all drug evaluations (Prager et al., 2019). Power analyses can be performed using data from positive controls, pilot studies or previously published experiments. Investigators can use existing tools, such as the Experimental Design Assistant created by the National Centre for the Replacement, Refinement and Reduction of Animals in Research (Karp and Fry, 2021; Percie du Sert et al., 2017). Approaches that enable genotyping of live larvae without disrupting muscle integrity, such as the Zebrafish Embryonic Genotyper, can be used to ensure that a sufficient number of mutant and wild-type sibling larvae are examined in each replicate (Douek et al., 2021). For example, in a typical workflow, the zebrafish larvae obtained from a heterozygous incross are genotyped at 2 dpf, then drug or vehicle solutions can be added to the same number of wild-type or mutant larvae. Following this process, phenotypic assessments such as birefringence analyses and muscle function assays (discussed in detail below) are performed. The number of embryos and replicates needed may vary depending on whether the experiment is a large-scale screen or a validation study of a promising compound. Obtaining each replicate from different parental zebrafish is recommended to reduce any potential confounding effects due to genetic backgrounds.

### Drug compound sources

It is essential that the drug manufacturer data, such as company, catalog number and lot number, be reported for all zebrafish drug evaluations, as the same compound purchased from different commercial sources can have different pharmacological activity. In addition, chemical syntheses of compounds in large-scale drug libraries can vary between production batches and based on whether they are aimed at commercial or academic use (Coussens et al., 2018). Whenever possible, pharmaceutical-grade compounds should be used to ensure consistent purity and efficacy and to adhere to local veterinary and animal welfare guidelines.

### Drug dosage and timing

The dosing of compounds is an important consideration for drug screening as well as for targeted drug validation studies. In published studies of zebrafish muscle disease drug screens, the concentrations of small molecules applied to embryos have typically ranged from 1 to 33 μM (Tables 1 and 2). Concentrations greater than 50 μM can often elicit adverse events or off-target effects and are rarely used in high-throughput drug library screens in zebrafish (Jackstadt et al., 2022; Wittmann et al., 2012; Tran et al., 2007). When wild-type zebrafish embryos were used to evaluate the toxicity of a chemical compound library with a concentration range of 100 nM to 10 μM, most chemicals showed little or no adverse effect on embryo health and survival at 1 μM (Farr et al., 2020). Based on these findings, this study selected 1 μM as a drug screening dose in the *sapje* zebrafish model (Farr et al., 2020). Although it is recommended to use a standard dose across a library for a drug screen, in targeted drug validation studies, dosing should be determined empirically for each chemical, ideally through a dose-response test (Farr et al., 2020). Dose optimization may be performed for compounds that showed some toxicity at the screening dose or did not achieve the biomarker correction threshold determined in the experimental aims.

An issue related to drug dosing and toxicity is that compounds that are safe and well tolerated in mammalian preclinical models and humans may be toxic to zebrafish, and vice versa. One example is tamoxifen, which shows dose-limiting toxicity when given to embryonic and larval wild-type zebrafish (Yu et al., 2020). Compounds identified in screens as being non-selectively toxic, i.e. those that caused cellular or organ damage or death to both wild-type and disease-model fish, should be described when reporting drug screens. The information should include the dose, source of the compound, age of the fish when treated and duration of exposure. The ability to exclude toxic compounds early in the drug discovery process is a potential advantage of drug screening in the zebrafish model (Howe et al., 2013).

The timing of when drug compounds are applied to zebrafish is also an important variable. Drug screens using zebrafish NMD models have typically applied drug compounds at 24 h post fertilization (hpf) (Kawahara et al., 2011; Waugh et al., 2014). The 24 hpf time point is a convenient stage for dechorionating embryos and initiating treatments and, in the case of *dmd-*mutant zebrafish embryos, occurs prior to the onset of any obvious disease phenotype (Bassett et al., 2003). Additionally, morphogenesis is largely complete by 24 hpf, decreasing the need to exclude potentially teratogenic drugs from the screen. For some zebrafish muscle disease models, initiating treatments in much younger embryos or after the appearance of disease phenotypes may be appropriate. It is important to consider that drug dosage may need to be optimized to the developmental stage at which it is being used.

## Standards for phenotypic endpoints and outcome measures for zebrafish muscle disease models

One distinct advantage of the zebrafish system is the plethora of quantitative tools commonly used to assess muscle, myotendinous junction (MTJ) and neuromuscular junction (NMJ) structure and overall function (Goody et al., 2010; Bailey et al., 2019; Granato et al., 1996). Skeletal muscle abnormalities in zebrafish can be evaluated via skeletal muscle birefringence, touch-evoked escape response and locomotion assays. These assays are non-invasive and high-throughput, and have, along with survival, already been described as the main outcome measures in previous large-scale drug screens in zebrafish. In each section and where relevant, we describe these endpoints and provide references to published protocols using these phenotypic assays.

### Muscle birefringence

One of the most commonly used assays of muscle structure is birefringence. Birefringence results from the diffraction of polarized light through the organized sarcomeric form, thereby illuminating the axial muscle of zebrafish larvae (Smith et al., 2013). Unaffected or wild-type zebrafish larvae produce bright birefringent muscle. In contrast, zebrafish larvae with abnormal muscle structure, such as the dystroglycan (*dag1*)-deficient zebrafish that model congenital myopathy, have generally reduced birefringence, and zebrafish larvae with dystrophic or damaged muscle often display darkened patches or holes (Smith et al., 2013; Berger et al., 2012). One advantage to birefringence imaging is that the entire larva can be imaged on a stereoscope, thus allowing the acquisition of large sample sizes (Berger et al., 2012). Birefringence data can be normalized and quantified to allow comparisons of the relative change in muscle structure over time and between zebrafish strains, comparisons of the treatment effects of different drugs, and comparisons and validations of treatment effects between different laboratories (Farr et al., 2020; Kilroy et al., 2022). Examples of skeletal muscle birefringence have been described for the *sapje* and *softy* (*lamb2*) mutant zebrafish, which have unique patterns of skeletal muscle birefringence that can be used as a biomarker for drug screens (Bassett et al., 2003; Jacoby et al., 2009) (Table 2). Muscle birefringence in zebrafish phenotypic evaluations can be employed in two ways: (1) to differentiate between the wild-type and affected zebrafish and (2) to determine the therapeutic efficacy of drug molecules in correcting or ameliorating the phenotypes of zebrafish NMD models. Of note, because larvae can exhibit variable birefringent brightness based solely on their positioning under the lens, consistent positioning of the fish for measuring muscle birefringence is critical (see references in Table 2).

### Touch-evoked escape response

Wild-type zebrafish show locomotive phenotypes very early in their embryogenesis, such as contraction of tail muscles by 24 hpf and organized swimming patterns by 48 hpf (Li et al., 2017). Touch-evoked escape response can be used to evaluate muscle function. This assay primarily focuses on the swimming behavior and overall movement of dechorionated zebrafish larvae upon tactile stimulation. Tactile stimulation, with a probe or similar physical stimulus, causes a brief burst of locomotion due to the contraction of fast-twitch muscle fibers (Smith et al., 2013). Upon such external stimulation of the muscle, wild-type zebrafish larvae at 2 dpf exhibit a pronounced escape contraction with a uniform swimming pattern. Conversely, zebrafish muscle disease models often show slow swimming behavior with a modest escape contraction and weakened locomotion (Sztal et al., 2016; Saint-Amant and Drapeau, 1998). An example of this is demonstrated in the Charcot–Marie–Tooth disease zebrafish model, in which *DNM2^S619L^* transgenic zebrafish display paralysis after touch-evoked stimuli compared to wild-type non-transgenic sibling controls (Zhao et al., 2019a). These locomotive assays are quantitative and exhibit several advantages, including the ability to unobtrusively monitor the NMD progression of many fish in a short time period (Widrick et al., 2016). A necessary experimental consideration is that larvae may require more than one stimulus before initiating the escape response, and tactile stimulation can mechanically damage some larvae. Increased experimental sample size or frequency of stimulation may overcome these limitations; however, this must be balanced with the consideration of the fragility of the zebrafish muscle mutant to avoid inflicting stress on the larvae.

### Locomotion tracking

Locomotion assays are helpful in testing muscle performance of zebrafish. These assays can record muscle performance over a prolonged period and overall locomotor activity in developing zebrafish larvae. The swimming behavior of wild-type or mutant zebrafish can be monitored through a visual tracking system measuring the mean distance, velocity and overall activity, thereby estimating skeletal muscle locomotor function (Zhao et al., 2020). Before 6 dpf, embryos and larvae have sustained periods of inactivity, which can confound the measurement of locomotion. One strategy to overcome this is to supplement the fish embryo medium with optovin, a small molecule that can induce locomotion upon photo-activation, prior to recording (Kokel et al., 2013). Upon light exposure, optovin-treated zebrafish with an intact sensory nervous system initiate rapid swim responses that can be easily measured and quantified (Sabha et al., 2016). After 6 dpf, free swimming can be captured without a chemical precipitant. Evaluating locomotion, including swimming behavior, is essential to examine the phenotypic outcomes of any given zebrafish model in any drug screening and high-throughput mutation-based studies that involve skeletal muscle (Sztal et al., 2016). These swimming behavior assays can be quantified for corrective outcome changes and drug advancement of lead therapeutics (Widrick et al., 2023).

### Considerations for pleiotropic phenotypes in zebrafish models of NMD

Pleiotropic phenotypes, such as neurological or cardiac defects, could represent meaningful outcomes of NMD drug testing and also confound locomotor behavioral testing. For example, congenital muscular dystrophies, particularly dystroglycanopathies, affect the brain and eye in addition to the skeletal muscle (Nickolls and Bönnemann, 2018; Bouchet-Séraphin et al., 2015). Seizures are common in patients with dystroglycanopathy and can be easily captured in zebrafish using quantitative video tracking in the presence or absence of the convulsant pentylenetetrazole. Seizures have been successfully used as the key phenotypic readout in high-throughput screening of zebrafish models of genetic epilepsies to identify novel drug targets (Baraban, 2021). As seizures are often recorded as increased activity and stereotyped movements, additional measures independent of motor function, such as electrophysiological recordings, should also be used. Newer technologies such as electron impedance myography can be adapted to help distinguish neurological disorders from muscle phenotypes in zebrafish models of disease in a high-throughput fashion (Cho et al., 2015; Rutkove et al., 2023). Consideration for secondary factors that influence movement and motor function, such as the development of central or peripheral nervous system lesions, may affect swimming behavior and should also be reported and addressed prior to the screening (Ohnmacht et al., 2016; Burris et al., 2021).

Cardiac pathologies can be addressed in zebrafish as these phenotypes often arise in patients with NMD and because many skeletal muscle proteins are also expressed in cardiac muscle (Nayak et al., 2023; Steffen et al., 2007; Li et al., 2017). However, many technical and experimental challenges can confound interpretations of these findings. For example, although pericardial edema is often associated with cardiac dysfunction, the disruption of skeletal myofibers during early development may indirectly result in pericardial edema because hematopoietic progenitor cells circulate across the larval body, leading to potential misinterpretation of the experimental findings (North et al., 2009). Several reliable cardiac phenotypic assays can be used to quantitatively assess cardiac phenotypes and pathologies in zebrafish NMD models. Heartbeats per minute can be quantified visually using light microscopy, in addition to the zebrafish electrocardiography that can be performed in adult animals (Zhao et al., 2019b). Finally, cardiac histology and markers of hypertrophy can be quantitatively assessed in zebrafish NMD models, as this is a rapidly expanding field with new models emerging (Bowley et al., 2022).

Ocular phenotypes in zebrafish are also robust and quantifiable. Multiple techniques for screening vision deficits are well established for ophthalmological conditions and can be applied to drug screening for NMDs that present with eye involvement (Ganzen et al., 2017). The visual motor response is a startle response, elicited by both light exposure or light removal, that can be detected as early as 3 dpf and can be studied in the same system used for motor tracking (Ganzen et al., 2017). However, motor deficits can interfere with the visual motor response, and appropriate controls must be in place to differentiate a visual deficit from motor impairment (Emran et al., 2008). In parallel, visual deficits could contribute to optovin motor activation independently of the effect of the compound, and light-only controls must always be performed. Alternatively, the lower-throughput optokinetic response can be measured by automatically tracking eye movement following a moving stimulus in immobilized zebrafish (Brockerhoff et al., 1995). Some forms of congenital myopathy result in eye muscle weakness and ophthalmoparesis and are particularly amenable to study via the optokinetic response (Jungbluth et al., 2018). Dystroglycanopathies and, to a lesser extent, DMD are linked to retinal deficits due to the critical role of the dystrophin-glycoprotein complex in photoreceptor synapse function (Barboni et al., 2022; Hagedorn et al., 2023; Jahncke and Wright, 2023). These have not been extensively studied in the zebrafish, but lighting conditions and shifts in light-dark cycles could affect experimental outcomes as zebrafish have defined circadian rhythms (Wolter and Svoboda, 2020; Pintos et al., 2023). Given these considerations, it is essential to standardize lighting conditions and document any shifts in light-dark husbandry conditions for ocular pathologies that are reported in the zebrafish NMD model.

### Physiological force assessments

Physiological force assays measure the specific peak and eccentric contraction in zebrafish larvae and allow researchers to quantitatively assess the corrective effects of experimental drugs (Sloboda et al., 2013). In addition, the muscle cross-sectional area and sarcomere length can be measured and used as a reference parameter to normalize force measurements. The forces, such as active force and tetanic force, are defined according to the experimental force values. Applying external stressors such as requiring the zebrafish to move through a more viscous environment can also reveal subtle muscle phenotypes that might be normally missed through analysis in standard swimming conditions (Ruparelia et al., 2014; Prykhozhij et al., 2023; Lo et al., 2015). Myotomal evaluations of isolated muscle preparations have revealed that, with cross-sectional area normalization, isolated myofibers would produce less tetanic force in NMD mutants compared to wild-type when normalized for power (Mead et al., 2020). This approach works well for a small number of zebrafish and is more suitable for secondary validation of lead compounds owing to the more laborious nature of the isolated myofiber preparation protocols.

### Histological and cellular markers

Histological and immunological markers are often studied in zebrafish-based drug screening and basic molecular mechanistic studies. Many different muscle and NMD-related cell types express markers that can be used for labeling, and there are several transgenic zebrafish reporter lines with labelled skeletal muscle-related cells and tissues (Tesoriero et al., 2023). Here, we focus on some of the most commonly used markers (Table 3). The structure of skeletal muscle fibers can be readily observed by staining with phalloidin, which labels filamentous actin (F-actin) (Henry et al., 2005). Skeletal muscle differentiation can be visualized using antibodies recognizing different myosins and myosin-interacting proteins. The F310 antibody labels fast-twitch muscle myosins in zebrafish, whereas the MF-20 antibody recognizes light meromyosin and is a pan-muscle myosin marker (Bader et al., 1982; Hamade et al., 2006; Barresi et al., 2001). The F59 antibody recognizes myosin heavy chain and labels slow muscle fibers in developing zebrafish (Devoto et al., 1996). In mammals, the number and location of satellite cells can be assessed by antibody-based labeling of the muscle satellite cell markers Pax3 and Pax7 (Seale et al., 2000; Relaix et al., 2005). In zebrafish, Pax3a and Pax7a demarcate muscle progenitor cells originating from the dermomyotome that become established somites (Feng et al., 2006; Hammond et al., 2007). Muscle histology is frequently assessed in conjunction with other structures, such as MTJs or NMJs. The most commonly used antibodies for labeling MTJs recognize laminin, collagen, thrombospondin, periostin and fibronectin (Henry et al., 2005; Kudo et al., 2004; Pagnon-Minot et al., 2008; Snow and Henry, 2009). MTJ quantification includes analysis of MTJ angles, concentration of MTJ proteins and the percentage of broken MTJs (Snow and Henry, 2009). Researchers can assess the RNA expression levels of tendon markers *thbs4b*, *tnmd* and *col1a2* in zebrafish larvae MTJs (Chen and Galloway, 2014; Kague et al., 2019). For visualization of NMJs, fluorescently conjugated α-bungarotoxin is often used to label these structures and their alterations that occur in zebrafish NMD mutants (Gibbs et al., 2013).

**Table 3.**
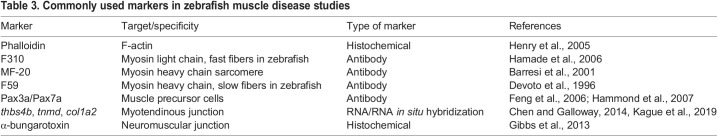
Commonly used markers in zebrafish muscle disease studies

Some zebrafish models of NMD also develop specific, disease-relevant histological phenotypes in their skeletal muscle tissues that are suitable for screening and have been used as outputs for testing targeted therapies. For example, zebrafish models of nemaline myopathy develop characteristic pathologic protein aggregates called nemaline bodies, which can be imaged using transgenic tagged proteins (Mansur et al., 2023). Similarly, zebrafish myofibrillar myopathy models develop BAG3^P209L^ aggregates that can be imaged by tagging them with eGFP, and zebrafish centronuclear myopathy models exhibit DNM2 aggregates (Ruparelia et al., 2021; Messineo et al., 2018; Gibbs et al., 2014).

Beyond skeletal muscle histology, anatomical markers can be used to analyze non-muscle phenotypes when these are expected, such as the aforementioned brain and eye anomalies in models of congenital muscular dystrophies and dystroglycanopathies (Karas et al., 2022 preprint; Liu et al., 2022, 2020). In addition, blood-brain barrier anomalies, which are characteristic in patients with LAMA2-related muscular dystrophy and are phenocopied in *lama2*-mutant zebrafish, are another potential phenotype that can be easily visualized using a histological dye-based assay (Gupta et al., 2012).

## Standards for addressing rigor and reproducibility

In addition to the standardization of drug screening parameters and outcome measures discussed above, zebrafish drug screening and drug treatment studies should adhere to a core set of study design, transparency and reproducibility standards (Landis et al., 2012; Prager et al., 2019). These address randomization, sample anonymization, sample size estimation and data handling. To allow for randomization, controls in drug treatment experiments should consist of zebrafish siblings. Importantly, if animal genotypes are determined using systems such as the Zebrafish Embryo Genotyper (discussed above) before drug testing, it is imperative that the allocation of animals of each genotype to control and treatment groups be randomized (Douek et al., 2021). The control and treatment sample labels should be masked from personnel scoring phenotypic endpoints, if possible. The drug screen is potentially most optimal if the samples are doubly anonymized, i.e. the experimenter is not aware of which group received the drug compound versus vehicle control and whether the zebrafish cohort is the mutant or wild-type control. We encourage researchers to engage a neutral party to perform this masking. This may be an independent laboratory or core facility that specializes in drug compound identification, screening and testing. To address sample size, power analyses should be performed. Biological replicates consisting of distinct sampling from individual zebrafish or independent spawns, not technical replicates in which sampling is replicated from the same animal or spawn, should be used. Additional controls, such as performing drug test replicates on different days with different fish stock tanks should also be performed if available. Additionally, positive controls that consist of treatment with known compounds that convey corrective benefits if known and negative controls, including vehicle-only treatments, should be part of all zebrafish drug screening.

Validation of candidate drug compounds identified in the primary screen, which are often referred to as ‘hits’, is essential to rigor and reproducibility in chemical screens. One way to validate a novel effect of a compound is to repeat the experiment with the same compound obtained from an alternative source. If available, validation of the results by a second group or laboratory, preferably an independent group, is encouraged. For example, two separate groups led by the authors of this article (Farr et al., 2020) previously independently validated a novel compound combination that ameliorates muscle defects in *sapje* zebrafish. When reproducing results from another group, experimental parameters and phenotypic readouts should be duplicated, as any deviation between protocols or experiments might confound interpretation of the results. Together, these steps ensure pharmacological reproducibility while removing elements of experimenter bias.

## Drug pharmacology and chemistry considerations

In this section, we discuss several issues related to the chemistry and activity of small molecules that should be considered when conducting drug treatments and drug screening in zebrafish. Although many of these are not frequently or easily addressed, greater attention to these issues will enhance the value and significance of zebrafish studies of small molecule therapies for NMD.

### Drug compound solubility

In the majority of reported drug screening studies, the compounds are either dissolved in fish water, usually kept at a salinity range of 0.25-0.75 parts per thousand (ppt), or in a mixture of dimethyl sulfoxide (DMSO) and fish water or embryo medium (Serafini et al., 2018; Lawrence, 2007). Only a few studies have reported oral administration of small molecules into the fish due to technical challenges such as toxicity, drug formulation demands and the potential for inconsistent drug dosing based on feeding amounts (Graham, 2016; Dang et al., 2016). Recently, a novel food-based drug pellet formulation was described for long-term drug treatment in adult BRAF-mutant zebrafish models of melanoma (Lu and Patton, 2022). This pellet formulation could be adapted for zebrafish NMD drug screens as investigators look to establish novel protocols for longer-lasting orally administered compounds.

The solubility of a molecule determines its absorption and resulting biological effects in the zebrafish (Lipinski et al., 2001; Long et al., 2019). Molecules with poor solubility in fish water or DMSO may be incorrectly classified as non-toxic or ineffective merely because they were not absorbed correctly. Hydrophobic moieties are first dissolved in DMSO and then diluted in fish water or embryo medium and applied in zebrafish assays. Of note, the percentage of DMSO should be maintained in the 0.01-1% range to minimize its cytotoxic effects (Crim and Lawrence, 2021; Bello et al., 2020). However, the risk of DMSO toxicity can vary with overnight or prolonged exposures and, in some instances, it may be preferable to modulate DMSO concentrations dependent on the assay (Baraban et al., 2013). It is also important to remember that higher concentrations of DMSO can affect autophagy, thus impacting cellular and whole-animal phenotypes (Song et al., 2012). Additional routes of compound delivery may be achieved via direct injection into the site of interest, including directly into the skeletal muscle. However, these methods may be technically challenging and the experimenter must consider the consequences of tissue damage upon injection and how this may affect the experimental readouts.

### Pharmacokinetics and pharmacodynamics

Gaining insight into the pharmacokinetic (movement of drugs through the body) and pharmacodynamic (the mechanism of action) profiles of small molecules in zebrafish can help guide validation and translation studies in mammalian models and potential clinical trials. Pharmacokinetic and pharmacodynamic parameters need more attention in zebrafish small-molecule treatment studies, particularly those concerning NMD. An unexplored area of zebrafish drug screening is the concept that larvae may reveal drug blood-brain barrier penetrance and influence complex neurobehaviors (Eliceiri et al., 2011). As wild-type zebrafish tend to have a lower metabolic rate than that of humans, humanized zebrafish that more closely match human metabolism may advance our understanding of pharmacokinetic profiles of small molecules in zebrafish compared to those in humans (Poon et al., 2017).

Indeed, an advantage of performing drug treatment studies in zebrafish is that pharmacological absorption, dissolution, metabolism, elimination and toxicity (ADME-Tox) properties can be evaluated in the whole organism (MacRae and Peterson, 2015; Patton et al., 2021). ADME-Tox assessments have thus far been lacking from drug treatment studies of zebrafish NMD models. However, previous reports in other areas have analyzed drug accumulation and metabolism in and elimination from zebrafish larvae using mass spectrometry-based approaches (Kantae et al., 2016; Kirla et al., 2018; Asslan et al., 2021). Alternative methods to determine drug uptake and distribution in zebrafish larvae include the use of radiolabeled or naturally fluorescent compounds (Souder and Gorelick, 2018). Assaying internal concentrations can provide valuable information on calculating various pharmacokinetic parameters to further translate drug screening findings to humans (Rennekamp and Peterson, 2015; Yadav et al., 2021). In particular, methods for nanoscale blood sampling in zebrafish larvae and pharmacokinetic-pharmacodynamic modeling demonstrated that the larval zebrafish response to an anti-tuberculosis drug could be translated to known effects in humans (Van Wijk et al., 2020). Despite these advances, measuring the biodistribution of compounds to specific organ systems in zebrafish embryos and larvae is difficult. Thus, determining precise drug levels in organs of interest has not typically been an aspect of drug discovery and development in zebrafish. Pigmentation changes occurring in the development of the zebrafish melanophores can impair light penetration and obstruct imaging of fluorochrome-conjugated drugs (Logan et al., 2006; Mizusawa et al., 2018). Nevertheless, better drug detection methods, such as improvements in drug conjugations with fluorochromes, advancements in microscopy and single-cell detection methods, may lead to the better evaluation of drug concentration levels in zebrafish tissues and cells.

### Toxic pharmacophore and functional groups

Understanding the adverse or off-target effects of approved or preclinical small molecules is a major hurdle in drug discovery and development. Off-target effects are one of the reasons behind the low percentage of drugs advancing to the latter stages of clinical trials (Poon et al., 2017). Similar to cell-based drug screening, the zebrafish is a useful model to provide toxicological details of test compounds, but with the added benefit of offering whole-organism testing. Identifying toxic pharmacophores (toxicophores) and functional groups in candidate drug compounds with the help of medicinal chemists may be highly useful for interpreting the results of a drug screen. Toxicophores and toxic functional groups in small molecules are often referred to as structural alerts. These structural alerts are chemical fragments that can be metabolized into highly reactive species or harmful reactivity entities. For example, carboxylic acid potentially forms a highly reactive acyl glucuronide, and the primary aromatic amine is oxidized into a hazardous *p*-quinone imine metabolite (Sun et al., 2022). These reactive chemical moieties are actively involved in the chemical reaction, ultimately leading to off-target drug compound binding. Chemicals can also be evaluated for toxicity by evaluating key liver cytochromes and LC_50_ values (Boelsterli and Kashimshetty, 2010; Limban et al., 2018; Bauer et al., 2021).

Taken together, the physicochemical properties of the small molecules are critical in determining the therapeutic effects of any experimental animal model. In 2001, Lipinski and colleagues reported experimental and computational approaches to predict the human oral bioavailability of drug molecules (Lipinski et al., 2001). These are now referred to as ‘rules of five’ or ‘Lipinski rules’ for absorption and permeation: the drug should have a molecular mass <500 Da, lipophilicity (logP) <5, fewer than five hydrogen bond acceptors and fewer than five hydrogen bond donors. Later updates to the Lipinski rules include additional parameters, such as a polar surface area of less than 140 Å and fewer than ten rotatable bonds (Veber et al., 2002). Notably, Long et al. (2019) evaluated whether drug compounds, when applied to zebrafish, followed these Lipinski rules to ascertain whether a compound is truly biologically inert or whether its inactivity is due to insufficient permeability into the model organism. The authors compiled a list of 700 chemical compounds that were used in published zebrafish assays and evaluated and compared their properties with the properties of US Food and Drug Administration-approved and known drugs. This comparison showed that zebrafish-active compounds had statistically significantly different molecular mass, lipophilicity, number of hydrogen bonds and polar surface areas compared to those of known drugs (Long et al., 2019). These findings, coupled with the updated Lipinski rules, can be useful for determining structure-activity relationships of promising drug compounds to facilitate their advancement to other animal models and eventually to patients.

## Conclusions and future directions

Of the commonly used animal models in biomedical research, the zebrafish has an optimal combination of advantages for elucidating the signaling pathways, the molecular mechanisms of different NMDs and the mechanisms of action of existing or repurposed drugs (Fig. 1). Drug screening and the discovery of potentially effective compounds is a challenging, time-consuming and expensive process. The transition from preclinical animal models to clinical trials often poses multiple hurdles as many parameters are involved.

We aim to improve the potential for translating small-molecule studies in the zebrafish model into patient therapies. Building upon the guidelines proposed for preclinical experiments in the *mdx* mouse (Willmann et al., 2012), we propose that preclinical drug screening and drug treatment studies in zebrafish NMD models should be conducted using the standardized approaches described above. The primary parameters that we have identified for the standardization of zebrafish NMD drug treatment studies are summarized in Box 1.

**Box 1. Primary parameters for zebrafish muscle drug screens**

**Details of animal care:** many variables of zebrafish husbandry are not yet completely standardized across laboratories and facilities. To facilitate future standardization of animal care, husbandry details, such as larval and adult diets, should be reported in publications in the Materials and Methods sections.

**Careful design of the experimental approach:** the choice of genetic strains used, adequate power, and drug treatment timing and dosage are all variables that should be considered and reported.

**Use of standardized protocols for common outcome measures:** outcomes for zebrafish muscle disease studies include birefringence and motility assays, which have quantifiable measures for comparing treatments within and between different laboratories.

In addition to the standard approaches and advantages presented here, some limitations of the zebrafish model remain. The age of the zebrafish at the typical time of drug testing is a notable limitation. Most studies have emphasized embryonic and larval time points, and drug screening studies on adult fish are rare (Babin et al., 2014). Similarly, most NMD drug screens in zebrafish have been initiated during embryonic stages, prior to 48 hpf. Thus, the zebrafish drug screening field still has a knowledge gap in muscle drug responses, muscle functional outcomes and long-term survival effects. Although motor phenotypes can be easily studied via automated tracking, the impact of co-morbid changes in neural tissues, particularly the eye, brain and spinal cord, that present in congenital forms of NMD throughout the lifespan, remains unknown. The lack of adult zebrafish model studies is partly attributed to challenges in administering drugs at scale to older fish and partly to the lack of high-throughput phenotyping capabilities. For example, birefringence assays are not possible in adult zebrafish as the assay requires optical transparency. However, the development of new techniques, such as polarization-sensitive optical coherence tomography, provides a promising tool for muscle imaging in adult zebrafish (Yang et al., 2020). Phenotype-based screening or mechanistic studies in embryonic, larval, juvenile and adult fish to investigate the effects of drugs throughout the lifespan of a disease model may further advance in the near future. As newer drug treatments for patients with NMDs emerge, the potential for combinatorial treatments to target previously unaddressed symptoms is now available. Drug screening in zebrafish NMD models may yield novel compounds that can be delivered to patients with NMDs to treat symptoms and pathologies that are inadequately tackled by single treatments. For example, there are now several disease-modifying therapies (e.g. nusinersen, onasemnogene, abeparvovec and risdiplam) for spinal muscular atrophy (SMA) that can extend lifespan, prevent motor neuron death and maintain ambulation in patients (Reilly et al., 2023). However, not all patients with SMA respond the same to these therapies and one can envision that zebrafish SMA models may be useful for identifying additional combinatorial drugs. Other rare diseases that have little commercial pharmaceutical interest due to lack of a sizable affected population may be modelled in zebrafish for drug screens and may be of interest to patient foundations or precision medicine groups.

In summary, we expect that the standardization of zebrafish drug screening established here will increase the likelihood that compounds identified in the screens will have future success in mammalian animal models, human cell lines and eventual clinical trials for patients with NMDs and other genetic disorders.
